# The Medical Student’s Vade Mecum

**Published:** 1858-02

**Authors:** 


ART. VIII.— *The Medical Student's Vade Mecum. A Compendium of Anatomy, Physiology, Chemistry, Poisons, Materia Medica, Pharmacy, Surgery, Obstetrics, Practice of Medicine, Diseases of the Skin, etc., etc.* By GEORGE MENDENHALL, M. D., Professor of Obstetrics and Diseases of Women and Children in the Medical College of Ohio, Member of the American Medical Association, etc., etc. Fifth Edition Revised and Greatly Enlarged. With Two Hundred and Twenty-four Illustrations. Philadelphia: Lindsay & Blakiston. 1857.

The work which we have before us, from Messrs. Lindsay & Blakiston, has now reached its fifth edition, a fact which is generally thought to be a sufficient indication of a favorable reception by the profession. It is now probably sufficiently well known to all, and it would be useless to go into an analysis of its pages, or a discussion of its doctrines. The author from the start, disclaims all attempt at originality, and hopes merely to have given a compendium of the most rational views on the several branches taught in our medical schools. The plan of the work is to present this information in the famillar form of questions and answers; and it is designed that this may serve to rescue the odds and ends of the student's time, from idle waste. We have simply glanced at the arrangement, and taken up, some of the subjects at random; and from our observation, can give testimony of the judicious manner in which the most important and salient points of the various branches have been selected. We do not suppose that this little volume is intended to be a companion to the practitioner, who of course, would not wish to read anything more brief, than the lectures from which he received his early instructions; but we conclude that whenever the venerable preceptor dismisses his student to the halls of medical learning, he gives him a quantity of good advice, and when he has concluded his admonitions on the snares and temptations of city life, the importance of preserving a *good moral character*, and devoting his mind exclusively to study, he mentions some books which it would be advisable for him to procure or carry with him. The Medical Student's Vade Mecum will be useful in many points of view, but we would add that it cannot supply the place of careful study of notes taken in the lecture room, the reading and investigating of cases, which nearly every student is able to see treated, and the reading of works which dilate on points which are merely touched upon by the professor.

The discussions which have lately arisen on a reform in the system of medical education, and in which the great majority have been in favor of legthening, if practicable, the term of medical lectures, have been induced by the difficulty of crowding into one course, a sufficiently elaborate consideration of the subjects which must be taught from a single chair. This is especially true of the practical branches, medicine and surgery; and in some instances, many important diseases are passed entirely without mention, to make room for a full consideration of others. These deficiencies should be filled up by the study of tolerably elaborate treatises, so that the student may become acquainted with the opinions of other men than those from whom he is receiving instruction; and the extensive field which a medical student has entered upon, precludes the possibility of learning by a mere recital of facts, but rather by a system of convincing reasoning, and of arguments in support of received theories and doctrines. Though the volume which we have under consideration evinces great industry and sound judgment in its compilation, we are convinced that it frequently does harm in excluding the more complete and elaborate works, a reference to which is necessary, in order to reap all the benefits which may be derived from a course of lectures; and we are confident, that in many instances information is forced into the brains of the student by these compendiums before examination, like the carbonic acid gas into a soda fountain, to come out with a great noise before the professor and leave the mind as innocent of medical knowledge as possible. That this has been used extensively by medical students, is shown by the fact, that in a comparatively short time, four editions have been exhausted. F.

